# Neuronal Migration and Lamination in the Vertebrate Retina

**DOI:** 10.3389/fnins.2017.00742

**Published:** 2018-01-09

**Authors:** Rana Amini, Mauricio Rocha-Martins, Caren Norden

**Affiliations:** Max Planck Institute of Molecular Cell Biology and Genetics, Dresden, Germany

**Keywords:** retina, lamination, neuronal migration, mosaics, connectivity

## Abstract

In the retina, like in most other brain regions, developing neurons are arranged into distinct layers giving the mature tissue its stratified appearance. This process needs to be highly controlled and orchestrated, as neuronal layering defects lead to impaired retinal function. To achieve successful neuronal layering and lamination in the retina and beyond, three main developmental steps need to be executed: First, the correct type of neuron has to be generated at a precise developmental time. Second, as most retinal neurons are born away from the position at which they later function, newborn neurons have to move to their final layer within the developing tissue, a process also termed neuronal lamination. Third, these neurons need to connect to their correct synaptic partners. Here, we discuss neuronal migration and lamination in the vertebrate retina and summarize our knowledge on these aspects of retinal development. We give an overview of how lamination emerges and discuss the different modes of neuronal translocation that occur during retinogenesis and what we know about the cell biological machineries driving them. In addition, retinal mosaics and their importance for correct retinal function are examined. We close by stating the open questions and future directions in this exciting field.

## Introduction

**Figure 1:**
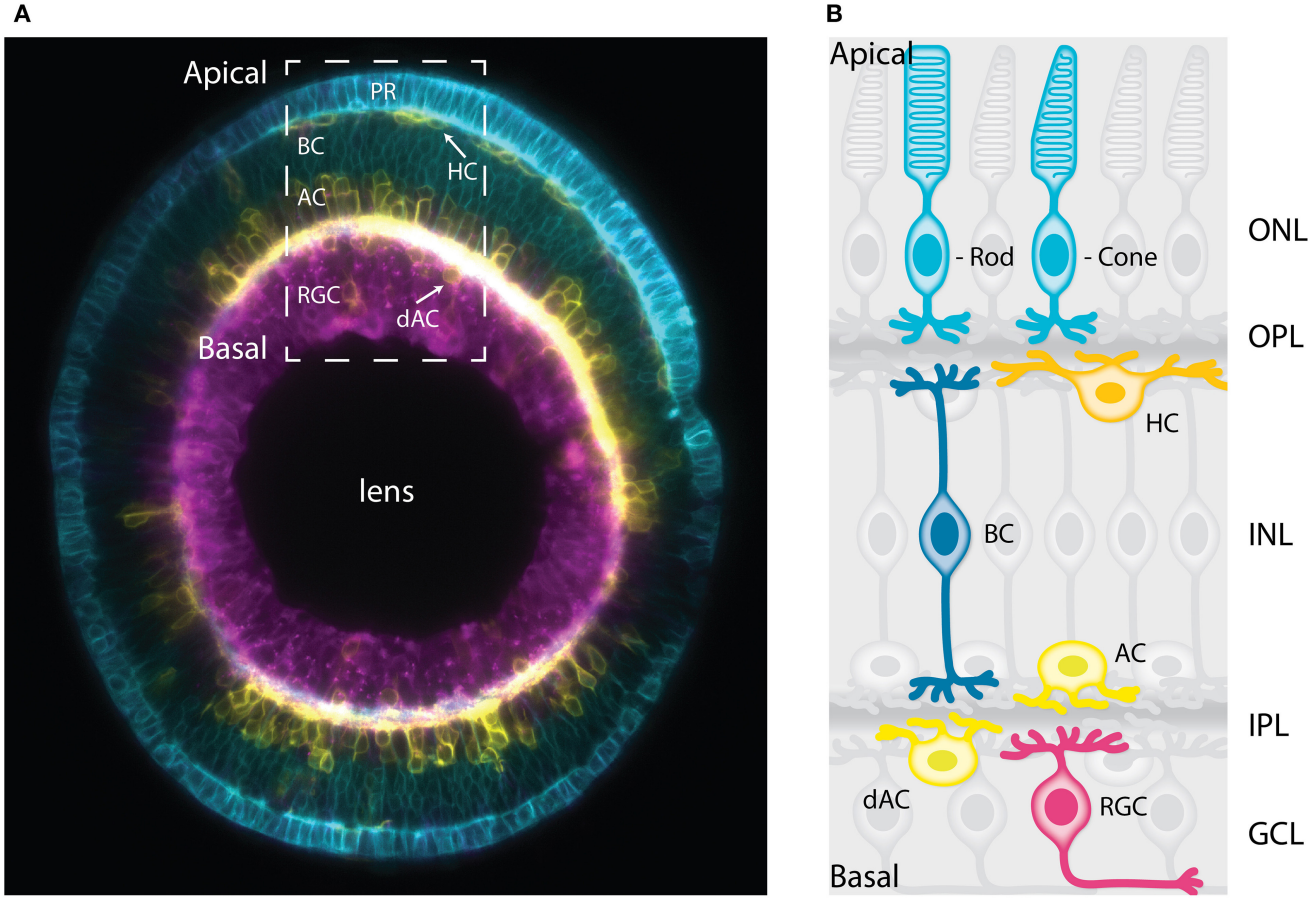
The vertebrate retina has a laminar organization. **(A)** Sagittal section of a mature zebrafish retina *in vivo*, showing lamination of cell bodies. The retinal cells are labeled with a combination of membrane-tagged fluorescent proteins that allows the identification of all the major neuronal types. In Cyan, the photoreceptors and bipolar cells under Crx promoter (Crx:gapCFP). In yellow, the horizontal cells, amacrine cells, and displaced amacrine cells under Ptf1a promoter (Ptf1a:Gal4/UAS:gapYFP). In magenta, the retinal ganglion cells under Ath5 promoter (Atoh7:gapRFP). Arrows indicate horizontal cells and displaced-amacrine cells. This image is a courtesy of Jaroslav Icha. The dashed box shows the area depicted by the schematic representation in **(B)**. **(B)** Schematic representation of a cross-section of the mature retina in zebrafish showing lamination of cell bodies and their neurites. The cell bodies of the retinal cell types are organized into three layers from apical to basal; the outer nuclear layer (ONL), the inner nuclear layer (INL), and the ganglion cell layer (GCL). These three layers are segregated by two plexiform layers enriched with axonal and dendritic processes, namely the outer plexiform layer (OPL) and the inner plexiform layer (IPL). The primary sensory neurons; rod and cone photoreceptors (cyan) are located at the most apical layer (ONL). The interneurons; horizontal cells (yellow), bipolar cells (blue), and amacrine cells (light yellow) are distributed along the apico-basal axis of the INL. The displaced amacrine cells (light yellow) and the output neurons retinal ganglion cells (magenta) occupy the most basal layer (GCL). PR, Photoreceptors; HC, horizontal cells; BC, bipolar cells; AC, amacrine cells; dAC, displaced amacrine cells; RGC, retinal ganglion cells.

The vertebrate retina is the part of the central nervous system (CNS) responsible for detecting, preprocessing, and sending visual information to the brain (Dowling, 1987). In this sense, it works as a processor that extracts relevant information from rich visual scenes (He et al., 2003; Wässle, 2004; Nassi and Callaway, 2009). To fulfill this function the retina has to be highly organized at the cellular and tissue level to allow the visual information to be sufficiently compressed, leading to fast transmission to the brain via the optic nerve (Nassi and Callaway, 2009; Sterling and Laughlin, 2015). In vertebrates, the retina is inverted, and light has to pass through the whole tissue before being collected by the photoreceptors. As a result, it was speculated that retinal architecture minimizes the number of neurons and wires that light has to pass through to avoid light scattering (Vos and Bouman, 1964; Hammer et al., 1995; Sterling and Laughlin, 2015). To solve such challenge in a limited volume, vertebrates established their retinal neural circuits by arranging the neuronal cell bodies into distinct layers in respect with their function (Figure 1B). This feature is called retinal lamination (Dowling, 1987; Hoon et al., 2014). This neuronal layer arrangement supports processing of the sensory signals (e.g., color, depth, and motion) in parallel. These are then analyzed in the different centers for visual perception in the cortex (Wässle, 2004; Werner and Chalupa, 2014).

Neuronal lamination is a hallmark of retinas across vertebrate species (Ramón y Cajal, 1893; Dowling, 1987) and disorganization of retinal lamination often leads to impaired overall organ function (Lahav et al., 1975; Duncan et al., 2011; Hoon et al., 2014). Despite the functional relevance of retinal lamination, we are only beginning to understand how this cellular organization is generated during development. We here provide an overview of how retinal lamination emerges across diverse species. We discuss the sequence of events necessary for the generation of a laminated retina and how these events are orchestrated. So far, migration of retinal neurons has been studied mainly in the zebrafish, chick and mouse models. However, since the birth order of retinal neurons is widely conserved between species, findings in one model organism can often be translated and compared to other species. In turn, differences between species provide the opportunity to learn about robustness and minimal parameters that are required to build a functioning visual system.

In this review, we first summarize the genesis of the different retinal cell types and discuss our current knowledge of how distinct neurons are placed in specific lamina via neuronal migration. In addition, the development of retinal mosaics is examined in light of their importance for the formation of functional neuronal units. We close by outlining open questions and future directions of retinal lamination at the cellular and tissue level. We concentrate on vertebrate systems as excellent literature on pattern formation in invertebrate retina already exists (Wernet and Desplan, 2004; Carthew, 2007; Cagan, 2009).

## Basic principles of neuronal lamination

Neuronal lamination is a common feature of the nervous system. Most areas of the CNS, including for example the neocortex and cerebellum, use layered neuronal arrangements as a strategy for information processing (Meunier et al., 2010; Guy and Staiger, 2017). The generation of a laminated brain structure can be achieved by different means which require different control mechanisms. During development, lamination of the CNS starts when neuroepithelial cells (NECs) enter differentiation programs. NECs follow a stereotypic sequence of cell cycle exit, cell-type determination, migration, and terminal differentiation. To generate a laminated and functional neuronal network, NECs need to give birth to the different neuronal types in the correct proportions at precise developmental stages. These cell types can be generated from a single pool (e.g., cortex; Malatesta et al., 2000; Anthony et al., 2004) or multiple pools of NECs (e.g., cerebellum; Miale and Sidman, 1961; Alder et al., 1996; Hoshino et al., 2005). The generation of different neuronal types often follows a stereotypic birth order (Donovan and Dyer, 2005; Kohwi and Doe, 2013). This birth order is implemented by either modulating competence and timing of differentiation or via cell intrinsic clocks (Stolt et al., 2003; Elliott et al., 2008; Alsiö et al., 2013; La Torre et al., 2013; Saurat et al., 2013) and/or exposure to extrinsic signals (Zhang and Yang, 2001; Rodriguez et al., 2012; Kohwi and Doe, 2013). The lamination timing can reflect the birth order of the neurons. For example, the six layers of the vertebrate cerebral neocortex are generated in a birth-date-dependent inside-out (apical to basal) manner, where early born cells occupy the inside layers and late-born neurons populate the more superficial layers (Cooper, 2008; Greig et al., 2013). In addition to this widely accepted view, recent studies suggest that the ultimate laminar fate of some neurons in the mouse neocortex is not completely determined at the time of birth but it is rather specified by the surrounding environment according to the positioning of the neurons within specific layers (Oishi et al., 2016).

Given that neurons are frequently born away from the position at which they later function, they have to move to their final layer through the developing tissue. Newborn neurons can take on complex migration routes to their final destination using different cytoskeleton components and modes of cell migration to navigate the developing tissue (Kriegstein and Noctor, 2004; Cooper, 2013; Icha and Norden, 2014). In addition, due to the fact that cell proliferation, migration and differentiation often occur in parallel and can thus influence each other, they need to be precisely orchestrated (Ge et al., 2006; Nguyen et al., 2006; Mairet-Coello et al., 2012; Rodriguez et al., 2012). For example, premature cell cycle exit during the early stages of development may disrupt laminar organization of neurons by increasing the relative number of early-born neurons at the expense of the neurons that constitute the layers formed at later developmental stages (Wang et al., 2011; Harrison et al., 2012). What we know about how these processes are regulated during retinal development will be discussed in the following sections.

## Building blocks of the retina

### Early retinal development from optic vesicle to neurogenesis

The retina originates from neural tube cells specified to form the eye field that is divided into two lateral domains that evaginate to form the optic vesicles (Fuhrmann, 2010). Each optic vesicle then invaginates to form a tissue of characteristic hemispheric shape, the optic cup (Fuhrmann, 2010; Kwan et al., 2012; Sidhaye and Norden, 2017). The external epithelial cell layer forms the retinal pigment epithelium, whereas the internal layer gives rise to the pseudostratified retinal neuroepithelium. Before differentiation onset, the neuroepithelium is composed exclusively of NECs that undergo symmetric proliferative divisions to expand the tissue. As development progresses, progenitor cells start to enter differentiation programs and produce postmitotic retinal neurons (Agathocleous and Harris, 2009).

### Retinal neuronal cell types, their position, and function

As the NECs leave the cell cycle and differentiate, the developing retina transforms into a stratified structure, containing six major types of differentiated neurons: retinal ganglion cells, the cone and rod photoreceptors, bipolar cells, amacrine, and horizontal cells (Figures 1A,B). Most of these cell types feature multiple subtypes that exert different functions and can be distinguished based on their morphology and transcription profile (Masland, 2012a; Macosko et al., 2015).

The cell bodies of the different cell types are found in specific nuclear layers and segregated from their axonal and dendritic processes which form the plexiform layers (Figures 1A,B). The most apical layer, the outer nuclear layer (ONL) hosts the photoreceptors responsible for collecting light from the environment (cones for color and rods for dim light) and providing the synaptic input to the outer plexiform layer (OPL; Lamb, 2013). The bipolar cells located at the medial part of the inner nuclear layer (INL) connect the OPL to the inner plexiform layer (IPL; Euler et al., 2014). The retinal ganglion cells located at the most basal layer (GCL, ganglion cell layer), collect the visual information from the bipolar cells and transmit it to the brain via the optic nerve (Sanes and Masland, 2015). Most amacrine cells are distributed along the basal region of the INL while a small subset resides in the GCL. Amacrine cells in the GCL are called displaced amacrine cells. Horizontal cells localize at the outer margin of the INL close to the OPL. Horizontal and amacrine cells extend processes in the OPL and IPL, respectively, that help integrate the visual message presented to the RGCs (Kamermans and Spekreijse, 1999; Thoreson et al., 2008; Grimes et al., 2010; Masland, 2012b). Müller glia are the only type of glial cells found in the retina. They span the entire thickness of the retina and provide mechanical support by gripping on the outer limiting membrane and the basal lamina (MacDonald et al., 2015). In addition, it has been proposed that they can act as optical fibers optimizing light passage from the retinal surface to the photoreceptor cells (Franze et al., 2007). They further provide trophic support to the retinal neurons which is vital to their health (Reichenbach and Bringmann, 2013).

### Committed precursors in the developing retina

**Figure 2:**
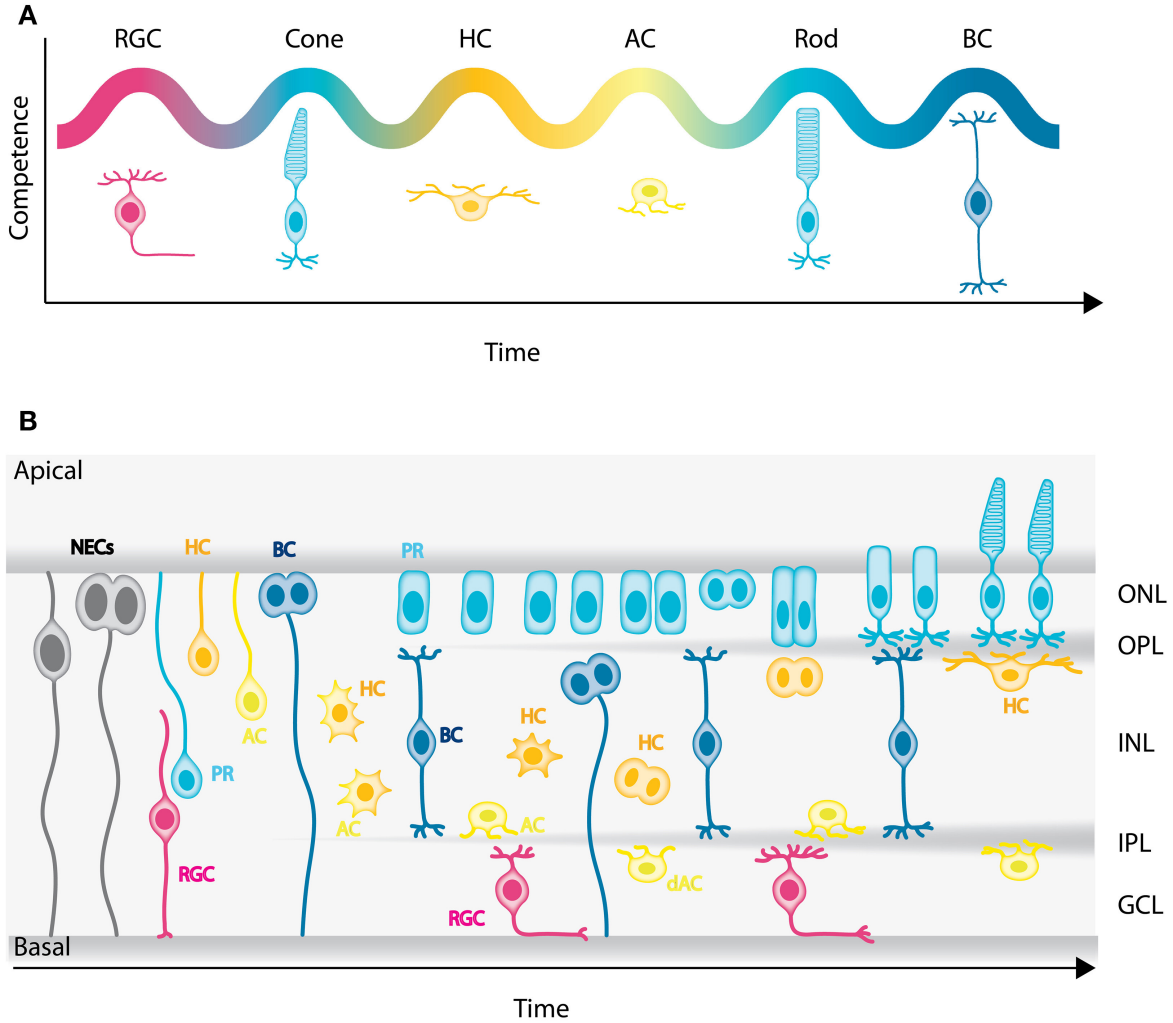
Genesis of the neuronal types of the vertebrate retina. **(A)** Chronological order of neuron birth in the vertebrate retina is depicted based on classical birth dating studies done across many vertebrate species. The first neurons born are the retinal ganglion cells, followed by cone photoreceptors, horizontal cells, amacrine cells, rod photoreceptors, and bipolar cells. Note that birth orders are overlapping and that we did not depict Muller cells in the schematic but they are the latest born cell-type. **(B)** Model of retinogenesis in the zebrafish embryo. Neuroepithelial progenitors (gray) divide asymmetrically at the apical side and give birth to one neuron either a retinal ganglion cell (magenta) or amacrine cell (light yellow) and one neuronal precursor committed to cone photoreceptor (cyan), horizontal (yellow), or bipolar (blue) cell fate. The committed precursors feature distinct morphology, expression of fate determinants and/or mitotic position. Cone photoreceptor precursors (cyan) show columnar epithelial morphology and divide within the developing photoreceptor layer at the apical surface of the retina. Horizontal cell precursors (yellow) are nonpolar and divide along the apico-basal axis of the INL, whereas bipolar cell precursors (blue) show bipolar morphology and can divide at apical or subapical positions. PR, Photoreceptors; HC, horizontal cells; BC, bipolar cells; AC, amacrine cells; dAC, displaced amacrine cells; RGC, retinal ganglion cells.

A single pool of multipotent progenitors gives rise to all retinal cell types (Turner and Cepko, 1987; Holt et al., 1988; Wetts and Fraser, 1988; Turner et al., 1990; Fekete et al., 1994). Classical birth dating experiments have shown that neurons are born in a stereotyped order that is conserved among vertebrate species including zebrafish, *Xenopus*, chicken, mouse, and rhesus monkey (Nawrocki, 1985; Young, 1985; la Vail et al., 1991; Belecky-Adams et al., 1996; Rapaport et al., 2004; Wong and Rapaport, 2009). The first neurons born are the retinal ganglion cells. Next, cone photoreceptors and horizontal cells emerge, followed by amacrine cells, rod photoreceptors, and bipolar cells (Figure 2A). Similar to cortical development, the timing of lamination in the retina reflects the neuronal birth order. However, in contrast to what has been shown in the cerebral neocortex, no strict relationship between the final laminar position and time of birth is observed in the retina.

Until recently, it was widely believed that, as retinal development progresses, NECs go through a deterministic series of competence states, similar to *Drosophila* neuroblasts (Cepko et al., 1996; Chen et al., 2012). This competence model was challenged recently however, by lineage analysis in rat and fish that suggest that stochastic mechanisms also play a role in the specification of NECs (Gomes et al., 2011; He et al., 2012; Boije et al., 2015). It was proposed that NECs commit to specific fates in a stochastic manner after their last apical division. However, terminal and penultimate divisions were biased toward particular fates, which cannot purely be explained by the stochastic model (He et al., 2012; Boije et al., 2015). One possible interpretation is that these divisions correspond to symmetric divisions of committed precursor cells. In agreement with this hypothesis, recent studies showed that a significant population of retinal neurons is generated by committed precursors, at least in zebrafish, chick, and mouse (Godinho et al., 2007; Rompani and Cepko, 2008; Hafler et al., 2012; Emerson et al., 2013; Suzuki et al., 2013; Cepko, 2014; Weber et al., 2014; Engerer et al., 2017). They can be distinguished from NECs by morphology, expression of fate determinants and/or mitotic position.

In zebrafish for example, it was shown that only the early born neurons, retinal ganglion cells, and amacrine cells, are exclusively generated by divisions of multipotent progenitors at the apical surface at early stages of retinogenesis. Later in development, cone photoreceptors, horizontal, and bipolar cells are born from symmetric divisions of committed precursors (Godinho et al., 2007; Suzuki et al., 2013; Weber et al., 2014; Figure 2B). Cone photoreceptor precursors show columnar epithelial morphology and divide within the developing photoreceptor layer (Figure 2B; Suzuki et al., 2013; Weber et al., 2014). Horizontal cell precursors are multipolar and divide either in the future INL or close to the future OPL (Godinho et al., 2007; Weber et al., 2014), whereas bipolar cell precursors show bipolar morphology and can divide at apical or subapical positions (Figure 2B; Weber et al., 2014; Engerer et al., 2017). So far, we are only beginning to decipher the origin and behaviors of committed precursors. Learning more about these particular progenitor types and how their emergence contributes and potentially facilitates retinal lamination will be interesting entry points for future studies.

## Neuronal translocation and lamination during retinal development

After the genesis of different neuronal cell types, the precise positioning of these neurons along the apico-basal (radial) axis of the retina is key for constructing the laminar architecture and subsequently functional neuronal circuits within the visual system. As such, neuronal migration is crucial for correct retinal layering. Given this, understanding how neurons migrate during retinogenesis is important to understand lamination and circuit formation.

### Cell biology of neuronal migration: modes and subcellular force generators

Neuronal migration has been most extensively studied in *ex vivo* cultures and organotypic slices of the cerebral neocortex and the cerebellum of rodents. The phenomenon of neuronal migration in the cerebral neocortex has been reviewed in depth elsewhere (Nadarajah and Parnavelas, 2002; Cooper, 2013; Icha and Norden, 2014; Hatanaka et al., 2016). Thus, here we only summarize key features of neuronal migration in the cerebral neocortex but focus on retinal neuronal migration and how it aids the generation of retinal wiring.

**Figure 3:**
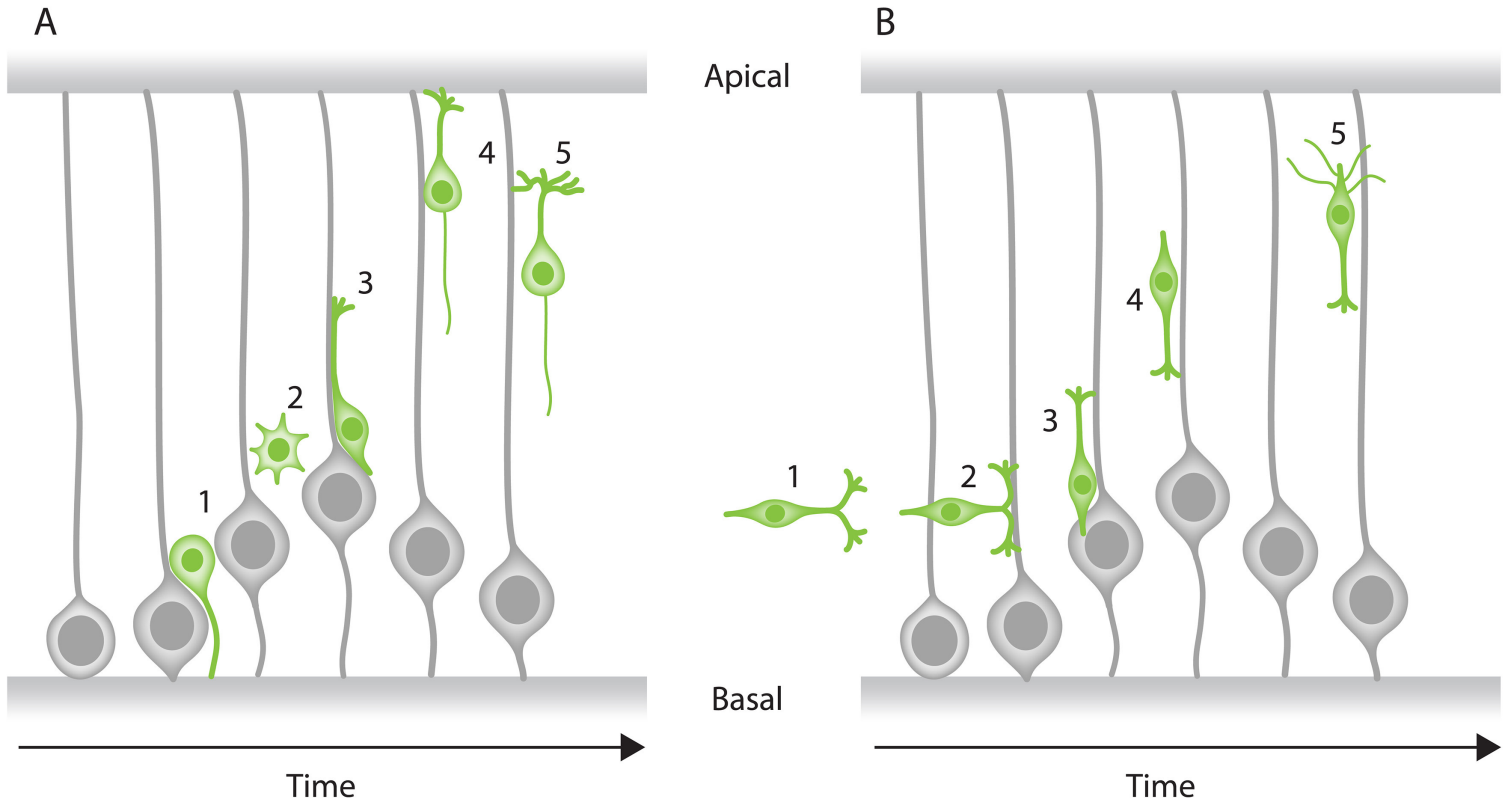
Modes of neuronal migration in the CNS. Two main modes of migration have been described in different parts of the nervous system: **(A)** Radial migration and **(B)** tangential migration. **(A)** A prevalent type of radial migration in the cortex is the glial-guided migration. It can be schematically summarized in three steps: (1) neurons born at the apical surface (2) lose their attachments to both the apical and basal surfaces of the tissue, (3) they attach to the radially oriented glial cells, (4) move along them to their target location perpendicular to the surfaces of the tissue and (5) undergo differentiation. **(B)** Some neurons combine radial migration with tangential migration to reach their target position. Tangentially migrating cells are not attached to the edges of the tissue and most of them form branched leading processes (arrowheads). As an example, tangential migration of interneurons is shown in three steps: (1) migration toward the cortex and move in parallel to the apical and basal surfaces, (2) interneurons subsequently associate with glial cells, (3) undergo radial movement along the glial-cells perpendicular to the apical surface and (4) reach their position and (5) undergo differentiation.

Traditionally, neuronal migration has been classified into two main modes: (1) radial migration and (2) tangential migration (Figures 3A,B). This categorization is based on the relative orientation of trajectories taken by the migrating neurons in the developing tissue. Radial neuronal migration means migration in parallel to the apico-basal axis of the tissue, while tangential migration is defined as neurons following a path perpendicular to the apico-basal axis of the tissue.

In the cerebral neocortex, radial migration is subdivided into two distinct modes: (a) somal translocation and (b) glial-guided migration (Figure 3A). At earlier stages of cortical development, neurons radially migrate within the tissue through somal translocation (Morest, 1970a; Nadarajah et al., 2001; Nadarajah and Parnavelas, 2002). Neurons migrating via this mode, exhibit either a unipolar or bipolar morphology and use their basal attachment to translocate their nucleus and other organelles (Nadarajah and Parnavelas, 2002; Cooper, 2013; Icha and Norden, 2014). As the cerebral neocortex thickens during development, some neurons attach to the radially oriented progenitor cells also known as radial glial cells. These neurons assume a bipolar morphology, with a leading (basal) and a trailing (apical) process and migrate along the glial cells to their target location (Rakic, 1971, 1972; Sidman and Rakic, 1973; Nadarajah and Parnavelas, 2002; Marín et al., 2010; Hatanaka et al., 2016).

Some neurons that later build the cerebral cortex move away from their point of origin via non-radial routes to reach their target position using a tangential migration mode (O'Rourke et al., 1992; Tan et al., 1995, 1998). Evidence for this mode of migration came from retroviral cell lineage tracing in rat and mouse, showing that clonally related neurons within the developing cerebral neocortex can be tangentially dispersed from their birth site (Price and Thurlow, 1988; Austin and Cepko, 1990; Walsh and Cepko, 1992; Reid et al., 1995). Most tangentially migrating neurons switch to radial trajectories and modes during their migration cycle to reach their final position (Figure 3B). Unlike the radially migrating neurons that have a single leading process, tangentially migrating neurons typically display both single and branched leading processes (Martini et al., 2009). Selective stabilization of one of these branches determines the directionality of the tangential migration (Martini et al., 2009). However, it is important to note that some tangentially migrating neurons (e.g., precerebellar neurons) exhibit a single leading process during their entire migratory cycle (Bourrat and Sotelo, 1990).

As described above, during both radial and tangential migration in the cerebral neocortex neurons are polarized and exhibit leading and/or trailing processes and thereby take on either a unipolar or bipolar shape. However, some neurons extend multiple dynamic protrusions into different directions, adopting a multipolar morphology. These neurons undergo a third mode of migration known as multipolar migration or “free migration.” Multipolar migration is characterized by frequent changes in direction and rate of migration as neurons move toward their final laminar position along either the radial or tangential axis of the tissue (Tabata and Nakajima, 2003).

In addition to cell morphology and orientation of migration, other parameters including speed, directional persistence, and cell biological machineries driving the migration define the neuronal migration modes. Moreover, it was shown that neurons within the CNS can dynamically change patterns of migration *en route*, depending on their type and location within the lamina (Tabata and Nakajima, 2003; Noctor et al., 2004; Marín et al., 2010; Faux et al., 2012).

The role of neuronal migration in building the distinguishing laminar feature of the retina has been acknowledged since seminal studies by Ramón y Cajal (1893). However, the precise cellular and molecular mechanisms of neuronal migration during retinogenesis remain elusive. This in part is due to lack of high-resolution time-lapse imaging of the developing retina. As such, so far, a rather descriptive understanding of retinal neuron migration was acquired. More recent studies are only beginning to get insights into the cellular machineries driving different migration modes. Some disparities exist between neuronal migration in the retina and the cortex. For example, in contrast to the cerebral neocortex, there is currently no evidence for radial glial guided-like migration in the retina. Considering that most retinal neurons migrate before the differentiation of Müller cells, glial-guided migration in the retina is probably unlikely. To fully exclude this possibility however, in-depth investigations of later developmental stages are needed. What is clear already is that different retinal cell types use different trajectories and migration modes to reach their destination within the retina. Below, we discuss the current state of knowledge of retinal neuron migration modes and their kinetics. We also summarize the intracellular components involved in these phenomena in vertebrates.

### Retinal ganglion cell translocation

**Figure 4:**
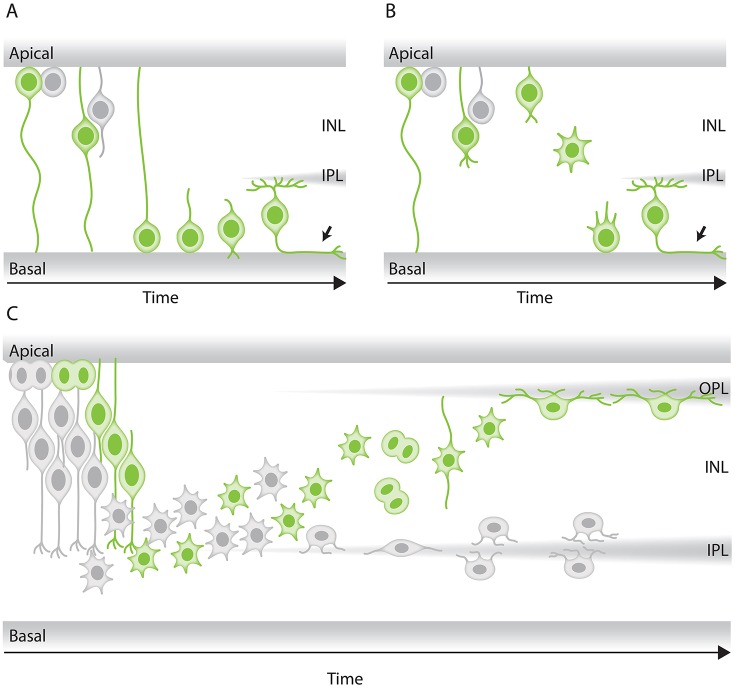
Modes of neuronal migration in the retina. **(A,B)** Scheme of different retinal ganglion cells (green) translocation modes: **(A)** somal translocation with basal process and **(B)** multipolar migration. **(A)** Retinal ganglion cells inheriting the basal process in zebrafish translocate basally faster than the sister cell. Basal translocation is followed by a period of fine positioning, during which cells lose their apical processes and project axons toward the optic nerve (depicted by arrows). **(B)** In rare cases in zebrafish, retinal ganglion cells lose their basal process, subsequently detach their apical process, increase their protrusive activity, and move basally in a multipolar migratory mode. The model shown is adapted from Icha et al. (2016) study. **(C)** Representation of retinal inhibitory neurons migration. Amacrine cells (gray) and the committed horizontal cell precursors (green) migrate to their laminar position via a combination of bipolar somal translocation and multipolar migration. Upon birth, they move away from the apical side using somal translocation. Later, they switch to a multipolar mode of migration and translocate their soma deeper into the INL. Amacrine cells remain at the basal INL positions, while horizontal cells revert their trajectory and migrate back toward the most-apical region of the INL, beneath the photoreceptor layer. On their way to the apical side, the committed horizontal cell precursors undergo mitosis with no positional preference along the INL. This model takes into account results from previous studies in the zebrafish retina (Weber et al., 2014; Chow et al., 2015; Icha et al., 2016).

Retinal ganglion cells are the first born retinal neurons. They undergo direct neurogenesis upon their apical division without an intermediate committed precursor step. Retinal ganglion cells then migrate away from their apical birth place to the most basal retinal layer adjacent to the lens (Figures 1A,B; Ramón y Cajal, 1893; Sidman, 1960; Nawrocki, 1985; Poggi et al., 2005; Zolessi et al., 2006). Retinal ganglion cell morphology and migration patterns have been extensively studied already decades ago using Golgi staining in the rat retina (Morest, 1970b) and by serial section electron microscopy in the mouse retina (Hinds and Hinds, 1974). Based on overall morphology of these fixed samples at different developmental time points, it was speculated that retinal ganglion cells move via bipolar somal translocation. Later studies, using live confocal fluorescence microscopy confirmed this migratory mode and showed that retinal ganglion cells translocate by moving their soma basally while remaining in contact with both apical and basal surfaces (Poggi et al., 2005; Zolessi et al., 2006). Recently, light-sheet imaging of the intact embryonic zebrafish retina provided more detailed insights into the retinal ganglion cell migration modes, kinetics, and mechanisms (Icha et al., 2016). The authors showed that the canonical mode of retinal ganglion cell migration is bipolar somal translocation. It was further uncovered that this somal translocation of retinal ganglion cells is accomplished in two phases: (1) a rapid directional phase, during which the retinal ganglion cell soma reaches the basal side of the retina and (2) a slower fine positioning phase within the retinal ganglion cell layer, which coincides with the loss of apical attachment that leads to random movements of the retinal ganglion cells within their layer (Figure 4A) most likely important for the exact positioning of cells. The fine positioning phase usually ends when axons of retinal ganglion cells start to grow (Icha et al., 2016). In addition, the authors unexpectedly found that retinal ganglion cells can also move by multipolar migration which is a much less frequent and less efficient mode of migration than the somal translocation (Figure 4B).

To understand force generation of retinal ganglion cell translocation, classic studies focused on the location of cellular organelles. Centrosomes, the Golgi apparatus, the primary cilium, and microtubules all reside in the apical process behind the nucleus of migrating retinal ganglion cells (Hinds and Hinds, 1974; Zolessi et al., 2006; Icha et al., 2016; Lepanto et al., 2016), making centrosome-based pulling an unlikely mechanism. Instead, the first phase of retinal ganglion cell migration in the zebrafish retina depends on an apically stabilized microtubule cytoskeleton and the inheritance of the basal process attachment (Icha et al., 2016). When either basal process attachment or microtubule integrity is impaired, retinal ganglion cells can switch to the multipolar migration mode that seems to lend robustness to the system (Figure 4B; Icha et al., 2016). Disturbing both migration modes results in a failure of retinal ganglion cell translocation and subsequently misplaced retinal ganglion cells (Icha et al., 2016). This in turn, disturbs future lamination events and retinal development as other later-born neuronal cell types are often ectopically placed within the retina. This implies that translocation of retinal ganglion cells sets the stage for all further retinal lamination programs in the zebrafish embryo (Icha et al., 2016). Overall morphological features of retinal ganglion cells in fixed retinal samples of mouse and rat suggest that retinal ganglion cells in other species also move by somal translocation (Morest, 1970b; Hinds and Hinds, 1974). However, these findings still need to be confirmed with live-imaging methods.

### Photoreceptor cell translocation

Photoreceptors are born at apical positions and also later reside at the most apical side of the retina (Figures 1A,B). In zebrafish, it has been shown that these cells go through a committed precursor stage and divide one more time apically, showing columnar epithelial morphology (Suzuki et al., 2013; Weber et al., 2014). As the birth-site and final residence of these cells are both at the most apical positions, it is possible that photoreceptors do not need to translocate much after their apical birth. However, studies using 3D human retina derived from embryonic stem cells reported that photoreceptors can be seen along the radial axis of the tissue (Kaewkhaw et al., 2015). In this study, live-cell imaging demonstrated that photoreceptors can slowly translocate their soma from the basal to the apical side during early stages of differentiation in 3D retinal cultures (day 42–44). Similar observations were made in the zebrafish retina wherein the photoreceptor precursors were detected at more basal locations before being seen apically before division (Suzuki et al., 2013). Whether such migration phenomena are common to photoreceptor precursors across species is not yet explored. In the future, it will be important to confirm this migratory mode and explore how it arises and how it contributes to retinal lamination.

### Horizontal cell and amacrine cell translocation

Horizontal cells and amacrine cells are inhibitory neurons occupying the INL. Horizontal cells lie at the most-apical (beneath the photoreceptor layer), while amacrine cells reside at the most-basal (just above the retinal ganglion cell layer) regions of the INL (Figures 1A,B). A body of classic studies using a combination of Golgi staining and electron microscopy demonstrated that migrating horizontal cells (Hinds and Hinds, 1979, 1983) and amacrine cells (Prada et al., 1987) can be seen with bipolar and multipolar morphologies. This has led to the suggestion that these cells could move via two different migration modes during retinal lamination. Amacrine cells have to move to basal INL positions, thus a behavior similar to that of retinal ganglion cells could be expected. While for horizontal cells, using the same rationale as for the photoreceptors, one would expect that they migrate only a short distance from their apical birth-site to their predestined position just slightly more basal.

In contrast to these assumptions, several studies over the past two decades provided evidence that horizontal cells, similar to amacrine cells, migrate substantial basal distances before returning to apical location where they later reside. First, analysis of the spatiotemporal pattern of horizontal cells showed that they are scattered along the radial axis of the developing retina in chicken (Prada et al., 1987), mouse (Liu et al., 2000), rabbit, cat, agouti, capybara (Silveira et al., 1989), and macaque (Wässle et al., 2000). This indicated that horizontal cell precursors undergo substantial migration after genesis and that this migration pattern may be evolutionarily conserved. Subsequently, live-imaging approaches in chicken (Edqvist and Hallböök, 2004) and zebrafish (Chow et al., 2015; Icha et al., 2016) confirmed that horizontal cells and amacrine cells both undergo basal migration and stop once they reach the prospective amacrine cell layer. Amacrine cells then remain in this layer, while horizontal cells revert their trajectory and migrate apically toward the future horizontal cell layer adjacent to the OPL (Edqvist and Hallböök, 2004; Godinho et al., 2007; Poche et al., 2007; Weber et al., 2014; Chow et al., 2015). These observations indicated that across species horizontal cells indeed undergo a bi-directional migration.

A detailed live-imaging study of the developing zebrafish retina focused on how horizontal cell precursors and amacrine cells reach the prospective amacrine cell layer. This work showed that horizontal cell precursors and amacrine cells reach their laminar positions using a combination of different migratory modes (Chow et al., 2015): first, they move from their apical birth-site to the INL while displaying bipolar morphology and directional persistence. Next, both cell types lose their apical attachment, switch to a less directionally persistent multipolar phase and translocate their soma deeper into the INL toward the prospective amacrine cell layer (Figure 4C). During the third phase, both horizontal cell precursors and amacrine cells undergo fine positioning via cell-type specific tangential migration. Amacrine cells move short distances and translocate deeper into the INL (Chow et al., 2015; Icha et al., 2016). Horizontal cells on the other hand start to demix from the amacrine cells and move toward the prospective horizontal cell layer and on the way undergo a final committed division before reaching their final position (Edqvist and Hallböök, 2004; Godinho et al., 2007; Poche et al., 2007; Shirazi Fard et al., 2013; Weber et al., 2014).

Overall, we do not yet understand how and by what intracellular mechanisms horizontal cells and amacrine cells reach their final location. Thus far, it was shown that fine-positioning of amacrine cells is influenced by the atypical cadherin Fat3 that cell autonomously influences the cytoskeleton and thereby the precise positioning of the dendritic arbor and the soma of developing amacrine cells in the mouse retina (Deans et al., 2011; Krol et al., 2016). In the case of horizontal cells, it was shown that demixing depends on the transcription factor *Lim1* (Poche et al., 2007). In conditional *lim1* knockout mice, horizontal cells fail to sort out from the amacrine cells and instead remain within the amacrine cell layer in the basal INL (Poche et al., 2007). However, how demixing and differential lamination of horizontal and amacrine cells is triggered remains unexplored. In the future, it will be necessary to carefully monitor horizontal cell and amacrine cell migration kinetics and trajectories and determine whether and how these two cell types influence each other during their migration. It will be also important to screen for intracellular and extracellular factors that might influence their migration and lamination.

### Bipolar cell translocation

Despite some insights on the regulation of bipolar cell fate specification (Livesey and Cepko, 2001), there is currently little information about the regulation of bipolar cell migration and laminar positioning. As mentioned previously, bipolar cell progenitors can divide apically or subapically depending on developmental stage of the retina (Figure 2B). Notably, the division plane of bipolar cell progenitors shows no preference for a particular orientation or position along the INL (Weber et al., 2014; Engerer et al., 2017). Recent studies also proposed that nuclei of bipolar cell precursors undergo interkinetic nuclear migration-like movement toward the apical surface of the developing zebrafish retina (Weber et al., 2014; Engerer et al., 2017). However, so far, we lack further information on how exactly bipolar cells that divide at different locations are positioned within the INL and how this positioning contributes to their neuronal function and circuitry.

### Future perspectives for understanding retinal neuron translocation

Overall, while some progress was made in recent years on neuronal translocation in the retina, this phenomenon and its underlying force generators are far from being understood. This needs further effort in future studies as only if the modes and kinetics of the different neurons as well as their interplay are understood can we begin to grasp how retinal lamination is achieved. As strong indications exist that different modes of migration are executed by different neuronal cell types and that some cells can switch migratory modes (Chow et al., 2015; Icha et al., 2016), we further need to understand the molecular mechanisms responsible for these changes. Accumulating evidence suggests that some modes of neuronal migrations outlined above are conserved across vertebrate species (Hinds and Hinds, 1974, 1979, 1983; Prada et al., 1987; Silveira et al., 1989; Liu et al., 2000; Edqvist and Hallböök, 2004; Godinho et al., 2007; Poche et al., 2007; Suzuki et al., 2013; Weber et al., 2014; Chow et al., 2015; Kaewkhaw et al., 2015; Icha et al., 2016; Engerer et al., 2017). Whether similar modes of migration also occur during human retinal development remains unclear. The recently developed human retinal organoids have the potential to become valuable model system to investigate these questions (Nakano et al., 2012).

## Extracellular players in retinal lamination

Neuronal migration plays a central role in building the laminar architecture of the visual system and it is thus important to understand not only the parameters that initiate but also the ones that appropriately terminate the migration of neurons. Many aspects of neuronal migration are still unexplored and it is unclear: (a) what guides distinct neurons that are born at similar locations and developmental stages, toward their differing layers and (b) how do neurons know when and where to stop migration.

It is clear that the gene expression profile of each neuronal cell type influences the cell's journey to its final destination. In addition, we have some insights on factors secreted by different cell types that could be involved in different aspects of retinogenesis from axon pathfinding (Li, 2005) to lamination (Fu et al., 2006). However, currently our knowledge is not sufficient to explain how retinal lamination is achieved. In the developing retina, neurons migrate in the context of neighboring cells and in an increasingly crowded environment, hence it is likely that they encounter contact-dependent cues from their surroundings. In response to such cues, neurons might modulate their physical features and adapt to those of their environment. This in turn might enable them to properly navigate within the tissue and reach their laminar destination. It is conceivable that these cues could either attract neuron types into their specific layers or repel them from integrating into inappropriate layers. Contact-dependent cues might be a source of positional information especially for neurons that undergo multipolar migration, particularly because they do not feature apically and/or basally attached processes that could provide them with directional information.

Contact-dependent cues could come from either cell-cell interaction or the interactions between cells and the extracellular matrix (ECM). The ECM has been shown to influence migratory phenomena in different contexts within the developing nervous system (Franco and Müller, 2011; Sidhaye and Norden, 2017). To test whether ECM also guides neurons in the developing retina, a first step would be to find whether analogous ECM components are involved in retinal neuronal migration as was shown in the cerebral neocortex (e.g., laminins, tenascins, and proteoglycans; Franco and Müller, 2011).

So far, only a limited number of studies have attempted to study extracellular influence on retinal neuronal migration and lamination. One of these studies showed that knocking down Laminin α1, a core component of the basal lamina, using morpholino approaches, impairs the basal translocation of retinal ganglion cells and results in their ectopic localization and subsequently a tissue-wide lamination defect in the zebrafish embryo (Icha et al., 2016). In addition, it was shown that the laminin receptor β1-Integrin is required in a neuron-autonomous manner for precise lamination of the ganglion cells in the mouse retina (Riccomagno et al., 2014). In the absence of β1-Integrin, the migrating ganglion cells do not stop upon reaching the laminin-rich inner limiting membrane and thereby form an ectopic retinal ganglion cell layer, phenocopying the retinal ganglion cell lamination defects observed in diverse laminin mutants (Edwards et al., 2010; Pinzón-Duarte et al., 2010).

In addition to ECM components, interactions between different cell types may also influence migratory behavior of neurons. For example, because of the sequential birth order in the retina one could speculate that the correct lamination of earlier born neurons could influence the subsequent layering events. However, this does not seem to be true in all cases. In the zebrafish lakritz mutant (Kay et al., 2001) and the mouse atonal homolog Atoh7 knockout (Brown et al., 2001), emergence of retinal ganglion cells is suppressed. Nevertheless, the remaining cell types are able to laminate correctly (Brown et al., 2001; Kay et al., 2001). In addition, INL and ONL still form even when retinal ganglion cell, amacrine cell, and horizontal cell fates are suppressed (Almeida et al., 2014). These studies suggest that neurons in the retina do not solely rely on the preceding neuronal types to migrate to their appropriate layer. However, how this is achieved and what guides different neuronal types to their correct layer, despite the lack of previously born neurons, remains elusive. In addition, it is important to note that several studies provide evidence that attractive or repulsive transmembrane guidance cues regulate correct stratification of neurites into IPL or OPL, across vertebrate species (Yamagata and Sanes, 2008; Matsuoka et al., 2011). Whether there is a mechanistic link between correct neurite stratification and termination of neuron migration and consequently lamination, remains to be investigated.

One re-emerging approach to study how retinal neurons interact with their neighboring cells and extracellular components are retinal reaggregate cultures. In early studies, reaggregate cultures were often used to understand how different tissues such as the chick retina are formed (Moscona and Moscona, 1952; Moscona, 1961; Layer and Willbold, 1993; Rothermel et al., 1997). In line with this, it was recently shown that the dissociated cells from the embryonic zebrafish retina, acquire a layered structure in a minimal culture condition, suggesting that they possess some intrinsic self-organizing ability to laminate (Eldred et al., 2017a,b). In the future, such *in vivo* reaggregate systems could provide a platform to investigate the nature of cell-cell and cell-ECM interactions required for retinal lamination. Moreover, manipulating the components of such *in vivo* aggregate systems could enable the simpler dissection of molecular and cellular mechanisms governing neuron migration, neurite stratification, and lamination within the retina.

## Linking lamination to retinal function

Visual perception is achieved through a series of processing steps that enable the retina to assess different aspects of the visual information. So far, we discussed how genesis of retinal cell types and their correct positioning contribute to the lamination of the retina. While these steps are prerequisites for proper retina development, building functional circuits is the next fundamental step for processing visual information and thus retinal function (Galli-Resta, 2000, 2001). The featured laminar design of the retina establishes various “functional units” across the radial axis of the tissue. Anatomically, each unit, depending on its function, is composed of different subsets of photoreceptors, bipolar cells, horizontal cells, amacrine cells, and retinal ganglion cells. However, each of these units detects and processes only a limited portion of the visual scene. To generate a holistic picture of the visual environment, the retina implements a third building rule: neurons of the same type are spaced in regular patterns within their respective layer, a phenomenon also known as retinal mosaics (Wässle and Riemann, 1978). Such retinal mosaics allow the complete sampling and a uniform coverage of the visual scene. In this section, we review our current knowledge on principles of assembly and maintenance of retinal mosaics in vertebrates.

### Emergence of retinal mosaics

**Figure 5:**
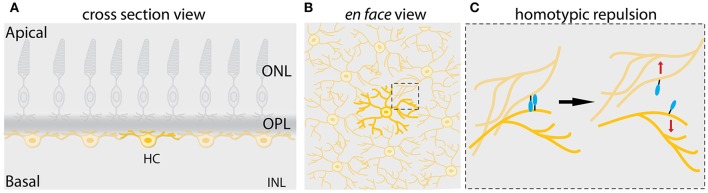
Mosaic assembly of horizontal cells in the vertebrate retina. **(A)** Schematic representation of a cross-section of the vertebrate retina showing that individual horizontal cells (yellow) are evenly spaced across the retina, a pattern known as retinal mosaics. **(B)** Schematic view from the surface of the retina in **(A)** showing that cell bodies and dendrites of horizontal cells of the same type are non-randomly distributed within the horizontal cell layer. Dendritic territories of the homotypic neighboring neurons show little or no overlap. This phenomenon is also referred to as dendritic tiling. The dashed box shows the area depicted in **(C)**. **(C)** Mosaic of horizontal cells arises by contact-mediated repulsion among these neurons. Horizontal cells of the same type express the same cell-surface molecules (depicted in turquoise). This allows the homotypic neurons to recognize each other and generate contact-dependent repulsion between their dendrites (depicted by red arrows), thereby creating mosaic spacing of horizontal cell soma. This figure takes into account results from previous studies in the vertebrate retina (Wässle and Riemann, 1978; Scheibe et al., 1995; Huckfeldt et al., 2009; Kay et al., 2012).

The highly ordered mosaic architecture in the vertebrate retina ensures that cell bodies of the same neuron-types, also called homotypic neurons are distributed in non-random arrays within their respective layer and at any given location in the retina (Figure 5A; Reese and Keeley, 2015). For example, the seminal study by Wässle and Riemann provided evidence that cone photoreceptors, retinal ganglion cells and horizontal cells in cats and monkeys are all arranged in a way that the distance between cell bodies of neighboring homotypic neurons is uniform and significantly different than that of random points (Wässle and Riemann, 1978). In addition, the arrangement of dendritic arbors of the homotypic neighboring neurons within an array are also spatially regulated so that they show little or no overlap, a phenomenon known as tiling (Figure 5B; Reese and Galli-Resta, 2002; Reese and Keeley, 2015). Although the retinal mosaic patterns are known to be fundamental to the processing of visual information in the retina, when and how neuronal mosaics emerge during development is not yet completely understood. One reason for this gap of knowledge is the fact that most existing markers are not expressed in neurons prior to their entry into their arrays during development.

Studies in different species, ranging from fish to primates, have suggested that retinal mosaics emerge early during development, at times when neurogenesis, migration and lamination are still in course. In fact, cone photoreceptors, horizontal cells, and cholinergic amacrine cells across species form regular arrays even when layering and polarization is not yet complete (Larison and Bremiller, 1990; Raymond et al., 1995; Scheibe et al., 1995; Galli-Resta et al., 1997; Novelli et al., 2005). Yet surprisingly, the geometry of the mosaic remains unaltered throughout the time new neurons enter the previously assembled networks. This implies that (a) regular spatial arrangement is a cell intrinsic property of the retinal neurons and that (b) their mosaic regularity is maintained during development.

Initially, it was assumed that the position of retinal neurons is fixed along their radial axis from their birth site. This led to the hypothesis that the spatial regularity of the retinal mosaics is due to fate determination mechanisms that act around the time of neuronal birth (Reese and Galli-Resta, 2002). Interestingly however, several lines of evidence, mainly using lineage tracing techniques demonstrated that particular types of differentiating neurons (e.g., retinal ganglion cells, horizontal cell, cone photoreceptors, and some type of amacrine cells) are often laterally displaced from their columnar clone of origin in the developing retina (Reese et al., 1994). The key observation confirming tangential dispersion came from a clonal territory analysis in X-chromosome inactivated transgenic mice (only half of the retinal progenitors are marked). There, it was shown that certain differentiating neurons including cone photoreceptors, amacrine cells, horizontal cells, and retinal ganglion cells tangentially move away from one another possibly to generate spatial order within mosaic arrays (Reese et al., 1995). Furthermore, using chimeric mice, it was revealed that the amount of this tangential dispersion varies depending on cell type (Reese et al., 1995, 1999). While the existence of tangential dispersion does not necessitate its direct contribution in forming mosaic arrays, following the cholinergic amacrine cells from their birth showed that their mosaics are assembled and maintained via tangential dispersion in mouse retina (Galli-Resta et al., 1997). Further, combining experiments with mathematical modeling showed that the spatial regularity of cholinergic amacrine cells is dynamically preserved throughout the period of new amacrine cell addition to a previously assembled array (Galli-Resta et al., 1997). This implies that amacrine cells within a mosaic array actively disperse to accommodate the new amacrine cells inserted into their mosaics. In the current view this means that tangential migration is a local phenomenon that contributes to building the mosaics of a particular subset of neurons (Cook and Chalupa, 2000; Galli-Resta, 2002; Reese and Keeley, 2015).

Some effort has been made to understand what drives tangential dispersion during mosaic assembly and maintenance. One proposed mechanism is that either diffusible signals or contact-mediated interactions between homotypic neurons account for tangential dispersion of neurons and subsequently mosaic regularity of neurons (Reese and Galli-Resta, 2002). This would suggest that a local spacing rule keeps a minimum distance between immediate homotypic neighbors in mosaic arrays. Consistent with this, mathematical modeling and simulations showed that a simple minimal spacing rule termed “exclusion zone model” ensures that neighboring homotypic cone photoreceptors in the macaque retina are distributed with a fixed minimal distance to each other and that they do not reside closer than that distance (Shapiro et al., 1985; Eglen and Willshaw, 2002). Similar conclusions were drawn from other computational studies seeking to simulate the role of the minimal spacing rule in controlling mosaic formation of other retinal neuron types across different species (Cook and Chalupa, 2000; Galli-Resta, 2002). Biologically, the minimum spacing rule implies that homotypic neurons repel each other and take appropriate distances from one another to keep their orderly array. Early evidence showed that emergence of horizontal cell dendritic neurites coincides with their tangential dispersion in mouse retina (Reese et al., 1999). This observation led to the hypothesis that dendritic interactions of homotypic horizontal cells might trigger their tangential dispersion and consequently mosaic formation. Using modeling techniques, this hypothesis was further investigated (Eglen and Willshaw, 2002), but a biological evidence for the role of dendrites on mosaic formation came from studying mosaic arrays of cholinergic retinal ganglion cells in the mouse (Galli-Resta et al., 2002) This work showed that disruption of microtubules in dendrites of the cholinergic retinal ganglion cells results in a reversible disruption of their mosaic regularity (Galli-Resta et al., 2002). Subsequent studies showed that horizontal cells achieve their mosaic arrangements via homotypic short-ranged interactions of their developing dendritic arbors in the mouse retina. These interactions are repulsive and enable homotypic neighboring horizontal cells to spatially arrange their soma (Huckfeldt et al., 2009), suggesting that a molecular cue exists that allows for a homotypic cell-type-specific recognition which in turn orchestrates the cell-cell contact-dependent repulsion of homotypic neurons and their mosaic patterning (Figure 5C). Direct evidence for the existence of such a cue came from a study in the developing mouse retina. Using a combination of microarray and gene knockout analysis, the authors found that signals initiated by two transmembrane proteins, MEGF10 and MEGF11 mediate homotypic interactions among horizontal cells and starburst amacrine cells and reposition their soma (Figure 5C; Kay et al., 2012).

Despite some progress, the exact mechanisms by which the homotypic neurons that form mosaic arrays reliably recognize each other while migrating in the pool of diverse neuron types in the vertebrate developing retina remain elusive. Besides tangential migration, programmed cell death and cell-fate lateral inhibition have been also proposed to contribute to building mosaic arrays within the developing retina (Reese and Keeley, 2015). Nevertheless, to what extent they interplay with tangential dispersion and promote retinal mosaic formation across vertebrate species is not yet understood. Finally, other parts of the CNS also exhibit regular arranged neuronal arrays (Cook and Chalupa, 2000; Budry et al., 2011). Therefore, in the future it will be interesting to examine similarities and disparities of spatial organization of neurons in different regions of the CNS and study whether and how such spatial regularities contribute to CNS development and function.

## Conclusions

Neuronal lamination requires the regulation of a well-orchestrated chain of events involving the generation of the right types and number of cells, positioning them at the correct places through migration and finally integrating them into their functional circuitries. Defects in the proper execution in any of these steps may result in neuronal layering defects and consequently impaired retinal function. Despite recent progress, we still lack an overarching picture of how different aspects of lamination from neuronal birth to final positioning occur at the cellular and tissue scales. This is partly due to the fact that most studies investigating neuronal lamination in the retina relied on fixed samples and conventional confocal microscopy that implies high phototoxicity leading to short observation times (Icha et al., 2017). Recently, light sheet microscopy enabled scientists to discover an unforeseen mode of migration for retinal ganglion cells that would not have been discovered with more conventional techniques (Icha et al., 2016). Thus, employing the rapidly advancing imaging tools to understand the behavior of retinal neurons from birth to differentiation will help to generate a more-detailed understanding of lamination in the vertebrate retina. The zebrafish retina due to its fast development, translucency and availability of genetic and molecular tools, has been a prominent model for retinal development. As some important features of retinal lamination are conserved across vertebrate species this understanding is most likely widely applicable.

It is known that the process of lamination is regulated by a combination of intrinsic and extrinsic influences during development. We highlighted the potential role of ECM in neuronal migration, mosaic formation and lamination which needs to get into the spotlight in future studies. Another important question is to what extent and how cell intrinsic and extrinsic cues interact and how they contribute to different aspects of lamination, from genesis to migration and mosaic formation, in the developing retina. As discussed in this review, communication between neurons could contribute to constructing the retinal layers and functional units, and the nature of such complex interactions *in vivo* needs to be investigated. In addition, the development of *in vivo* culture systems such as human organoids, is an exciting entry route to extract the conservation of diverse features of lamination. Remarkably, lamination is an organizational hallmark of many regions of the brain. As such, the retina as an outgrowth of the brain could provide a platform for a mechanistic understanding of neuronal migration and lamination in other laminated brain regions.

## Author contributions

All authors conceived and planned the review outline. RA and MR-M: wrote the initial manuscript. All authors read and edited the text and approved it for publication.

### Conflict of interest statement

The authors declare that the research was conducted in the absence of any commercial or financial relationships that could be construed as a potential conflict of interest. The reviewer OM and handling Editor declared their shared affiliation.
